# Metabolic Serendipities of Expanded Newborn Screening

**DOI:** 10.3390/genes11091018

**Published:** 2020-08-29

**Authors:** Raquel Yahyaoui, Javier Blasco-Alonso, Montserrat Gonzalo-Marín, Carmen Benito, Juliana Serrano-Nieto, Inmaculada González-Gallego, Pedro Ruiz-Sala, Belén Pérez, Domingo González-Lamuño

**Affiliations:** 1 Laboratory of Metabolic Disorders, Hospital Regional Universitario de Málaga, 29011 Málaga, Spain; 2 Instituto de Investigación Biomédica de Málaga-IBIMA, 29010 Málaga, Spain; javierblascoalonso@yahoo.es (J.B.-A.); montsegonzalo@yahoo.es (M.G.-M.); 3 Pediatric Gastroenterology, Hepatology and Nutrition Unit, Hospital Regional Universitario de Málaga, 29011 Málaga, Spain; serranonieto@hotmail.com; 4 Endocrinology and Nutrition Unit, Hospital Regional Universitario de Málaga, 29011 Málaga, Spain; 5 Department of Genetics, Hospital Regional Universitario de Málaga, 29011 Málaga, Spain; mcbenitol@gmail.com; 6 Unit of Metabolic Disorders, Centro de Bioquímica y Genética Clínica, Hospital Universitario Virgen de la Arrixaca, 30120 Murcia, Spain; inma.gonzalez@carm.es; 7 Centro Diagnóstico de Enfermedades Moleculares (CEDEM), Centro de Biología Molecular, Universidad Autónoma de Madrid, CIBERER, IdiPAZ, 28049 Madrid, Spain; prsala@cbm.csic.es (P.R.-S.); bperez@cbm.csic.es (B.P.); 8 Department of Pediatrics, University of Cantabria-University Hospital Marqués de Valdecilla, 39008 Santander, Spain; dglamuno@gmail.com

**Keywords:** newborn screening (NBS), incidental finding, dried blood spot (DBS), amino acids, acylcarnitines, next generation sequencing (NGS), hereditary metabolic disorders, inborn errors of metabolism

## Abstract

Incidental findings on newborn screening (NBS) are results that are not the target of screening within a given NBS program, but rather are found as a result of the screening and resulting diagnostic workup for that target. These findings may not have an immediate clinical impact on the newborn, but are sometimes an additional benefit of NBS programs and may be considered secondary targets of NBS programs. This work describes four case reports that had incidental findings on the NBS, which eventually led to the diagnosis of another metabolic disease instead of the one that was initially suspected. The first case was a new defect in the cationic amino acid transporter-2 (CAT-2), which was oriented as an arginase-1 deficiency in the newborn. The second case was a maternal glutaric aciduria type 1 (GA-1) that mimicked a carnitine transporter deficiency in the newborn. The third report was a case of lysinuric protein intolerance (LPI), which appeared as high levels of citrulline on the NBS. The fourth case was a mother with homocystinuria that was diagnosed during the biochemical study of vitamin B_12_ status. All cases provide new or interesting data that will help guide differential diagnosis in the future.

## 1. Introduction

The most important technological advance applied to newborn screening (NBS) in recent decades has been the introduction of tandem mass spectrometry for the early detection of inherited metabolic diseases [1]. This technology allows for the simultaneous measurement of amino acids and acylcarnitines in order to identify newborns at risk of suffering from certain aminoacidopathies, some organic acidurias, and fatty acid oxidation disorders. This is known as “expanded” NBS and it is estimated that up to 70 metabolic diseases and other clinical conditions can potentially be detected [2].

Classically, the objective of NBS programs has been to prevent the morbidity, mortality, and disabilities associated with the diseases that are screened for by initiating early, effective treatment. However, expanded NBS does not meet that goal for all detectable diseases. Some forms of disease may present in the first days of life (before the screening result is available), threatening the lives of newborns. Other forms of disease are lethal. For some of them, there is no effective treatment available. The natural history is not yet well known for all diseases and new, mild phenotypes continue to be discovered [3,4]. In any case, early diagnosis will usually prevent a diagnostic odyssey for the family; allow for genetic counseling; and, in many cases, improve the quality of life of affected infants.

Expanded NBS for inborn errors of metabolism is a promising field of targeted metabolomics. However, this new NBS model is highly controversial and has sparked international ethical debate for years, to the point that even today, many NBS centers in the world only report a limited number of diseases that must be evaluated with a higher level of scientific evidence in order to be included in the programs [2]. On the other hand, it is important to remember that all screened disorders are rare. Therefore, it is difficult to generate scientific evidence and it is practically impossible for a single NBS center to detect a sufficient number of cases of each disease to acquire enough experience. Therefore, international collaboration is necessary for the improvement of NBS programs [5]. 

If it is already difficult for an NBS program to decide which diseases to screen/report; the management of incidental findings is even more challenging [6]. Incidental findings on NBS are results that are not the target of screening within a given NBS program, but are found as a result of screening and the resulting diagnostic workup for that target. Laboratory personnel have a professional obligation to conduct a comprehensive evaluation of available test results to identify such clinically significant findings. These findings may not have an immediate clinical impact on the newborn, but are sometimes an additional benefit of NBS programs and may be considered secondary targets of NBS programs. The clearest example would be maternal vitamin B_12_ deficiency, which can be detected through screening for methylmalonic and propionic acidemia. Early identification of this condition and initiation of treatment are crucial in order to avoid potentially devastating and irreversible neurological damage [7]. Even if the disease detected has no treatment available, early detection would allow for adequate genetic counseling [8].

The objective of this work is to illustrate the importance of the diagnostic study of these incidental findings for newborns and their families through four case reports. These four cases share the peculiarity that the final diagnosis is an inborn error of metabolism different from the one initially suspected. Therefore, the term “incidental findings” that is usually used has been replaced by the authors with “metabolic serendipities”, in reference to the affected patients’ good fortune of these findings having been detected through NBS.

## 2. Material and Methods

All procedures were performed in accordance with the ethical standards of the competent committee on human experimentation (institutional and national). The patients or their legal representatives were counseled on and consented to DNA sequencing, which was performed in a certified reference laboratory as part of the patients’ clinical evaluation. All subjects gave their informed consent for inclusion before they participated in the study. The study was conducted in accordance with the Declaration of Helsinki of 1975, as revised in 2013, and the protocol was approved by the Ethics Committee of Universidad Autónoma de Madrid (CEI-105-2052).

### 2.1. Newborn Screening

Between April 2010 and December 2018, 368,152 dried blood specimens (DBS) that were collected at the third day of life from newborns born in Eastern Andalusia were analyzed during the newborns’ first week of life. The samples were prepared without derivatization from 2010 to 2014 and with derivatization using a commercial kit (MassChrom, Chromsystems, Munich, Germany) from 2015 to 2018. They were analyzed on an API3200 mass spectrometer (Sciex, Framingham, MA, USA) using an HPLC Series 200 System (Perkin Elmer, Turku, Finland). All amino acids and acylcarnitines were acquired in multiple reaction monitoring (MRM) mode. The cutoff values of these markers were established on the basis of the percentiles measured in the healthy population of newborns in their first week of life. The NBS for case 3 was performed at the Hospital Universitario Virgen de la Arrixaca (Murcia, Spain); her first DBS specimen was collected at 8 days of life.

### 2.2. Biochemical Confirmation Testing

Plasma and urinary levels of amino acids were analyzed using a quantitative method based on ion-exchange chromatography with post-column derivatization with ninhydrin (Biochrom, Cambridge, UK). Total plasma homocysteine levels were evaluated using a chemiluminescence immunoassay (Immulite 2000 system, Siemens Healthcare Diagnostics, Marburg, Germany). Urinary organic acids were measured as trimethylsilyl derivatives by gas chromatography/tandem mass spectrometry after urease treatment and ethyl acetate liquid–liquid extraction without oximation [9]. Plasma-free carnitine and acylcarnitines were quantified by a stable isotope dilution tandem mass spectrometry method. At the time of diagnostic confirmation, all mothers are routinely screened for amino acids and acylcarnitines in DBS to rule out maternal disease.

### 2.3. Molecular Analysis

Genetic analysis was performed on genomic DNA samples from patients’ whole blood. Highly pure DNA was extracted from whole blood using the MagNA Pure Compact Kit (Roche Applied Biosciences, Indianapolis, IN, USA) following the manufacturer’s instructions. An extended panel (Clinical Exome Sequencing, TruSight One Gene Panel (Illumina, San Diego, CA, USA)) that includes all known disease-associated genes (as of 2013) described in the Online Mendelian Inheritance in Man (OMIM) database (Mendeliome panel) was used. Following sequence enrichment, the readings were aligned with the reference genome hg19. All disease-causing variants reported in the Human Gene Mutation Database (HGMD) professional release 2020 database, the ClinVar database, and our in-house database, plus all variants with a minor allele frequency of less than 1% in the gnomAD and CSVS (CIBERER Spanish Variant Server (http://csvs.babelomics.org/)) databases were taken into account. Predictions of pathogenic variants were made using Alamut Visual software, which combines several predictors. Variants were classified according to American College of Medical Genetics guidelines [10,11]. Incidental findings in genes unrelated to the clinical/biochemical phenotypes were ignored, in accordance with European Union law. The libraries generated with the panel were sequenced using 150 bp paired-end reads using the Illumina NextSeq 500 next generation sequencing (NGS) platform. Candidate sequence alterations were confirmed by Sanger sequencing in the index case or in parent samples if they were available. The single nucleotide variants (SNVs) detected were confirmed by conventional Sanger sequencing. Mendelian segregation was done in the DBS for cases 1 and 3. To characterize a large deletion in the sample found in the infant who is case 1, we performed whole genome analysis with an 800 k single nucleotide polymorphism (SNP) array plus long-range polymerase chain reaction (PCR) using the Thermo Scientific Extensor Hi-Fidelity PCR Master Mix kit. RepeatMasker was used to characterize insertions.

## 3. Clinical Case Reports and Results

### 3.1. Case 1

Case 1 (reported in part by Yahyaoui et al., 2019 [12]). Male newborn from a controlled pregnancy without incidents. Eutocic vaginal delivery. Expulsion of meconium before 24 hours of life. Gestational age: 39 weeks. Birth weight: 2.260 g (<1st percentile). Birth length: 47 cm (4th percentile). Breastfeeding. Family history: healthy parents. He had a healthy 2-year-old sister. There was no consanguineous relationship between the parents or family history of interest.

On the NBS, he presented with slightly increased levels of arginine (67 µmol/L, normal value (NV): <33) and normal Arg/Orn. A second DBS specimen was requested and levels of arginine had increased to 112 µmol/L at 15 days of life. At that time, the infant was asymptomatic with good weight development and the main suspicion was a transient elevation of arginine or a mild form of arginase-1 deficiency. 

Plasma concentrations of cationic amino acids were high: arginine 312 µmol/L (NV: 34–88), ornithine 177 µmol/L (NV: 52–116), and lysine 599 µmol/L (NV: 199–209). The urinary amino acid profile was similar, but showed increased cysteine as well. The ammonia level was normal. 

Arginase enzyme activity in red blood cell extracts was normal (4796 µmol urea/h x g Hb; NV: 3471–7805) and no pathogenic variants were identified in the *ARG1* gene by Sanger sequencing. Having ruled out arginase-1 deficiency, we performed a clinical exome sequencing in order to find a new inborn error of metabolism related to the transport of cationic amino acids, or less likely, an arginase-2 deficiency. 

Exome sequencing revealed two loss-of-function variants on the *SLC7A2* gene: one small deletion in the maternal allele c.874delA (p.Ile292Leufs*2) and one large genomic rearrangement including exons 3 and 4 in the paternal allele. This gene encodes human cationic amino acid transporter 2 (CAT-2). At present, this is the only report of a defect in this transporter.

Currently, the child is 6 years old and is on a protein-controlled diet of 1.2 g/kg/day of protein with 30 g/day of protein- and amino acid-free formula to control amino acid levels. He presents with marked thinness, though he does not have any signs of malnutrition. He has been asymptomatic and presents with normal neurological and cognitive development, normal blood pressure, and no cardiovascular or immunological complications. 

### 3.2. Case 2

Male newborn from a controlled pregnancy without incidents. Eutocic vaginal delivery. Expulsion of meconium before 24 hours of life. Gestational age: 41 weeks. Birth weight: 4.830 g. Breastfeeding. Family history: healthy 21-year-old mother and 23-year-old father. He had no siblings. There was no consanguineous relationship between the parents or family history of interest.

On the NBS, he presented with an acylcarnitine profile compatible with carnitine transporter deficiency, with low levels of carnitine (C0), acetylcarnitine (C2), propionylcarnitine (C3), palmitoylcarnitine (C16), and stearoylcarnitine (C18), as shown in Table 1. Given the possibility that this metabolic disorder was of maternal origin, we performed a maternal screening for acylcarnitines on a DBS. The results of this test confirmed greatly decreased levels of C0, but also revealed elevated levels of glutarylcarnitine (C5DC), which made us suspect a maternal glutaric aciduria type 1 (GA-1) with a secondary carnitine deficiency (Table 1). When reviewing the newborn DBS results, it was striking that he had some slightly elevated ratios despite having normal levels of C5DC: C5DC/C8 6.72 (NV: <5.0) and C5DC/C16 0.14 (NV: <0.12).

On the maternal anamnesis, it was verified that her psychomotor development had been normal. The mother had completed compulsory secondary education and was currently working in the hotel/restaurant sector. During pregnancy, she had developed subclinical hypothyroidism and after delivery, she had anemia that required oral iron supplementation. She did not report any symptoms except asthenia. She had no exercise intolerance. She also had no headaches, tremor, or any other neurological symptoms.

The examination did not reveal macrocephaly. Her weight was 67 kg and height was 167 cm. Plasma C5DC was elevated (1.98 µmol/L; NV: 0.01–0.33) as well as urinary glutaric acid (1701 mmol/mol crea; NV: 1–4) and 3-OH-glutaric acid (106 mmol/mol crea; NV: 0–7). Plasma-free carnitine levels were considerably decreased (1.79 µmol/L; NV: 21–64). This biochemical study confirmed that she was a high excretory GA-1 patient.

The molecular study of the *GCDH* gene revealed the existence of two heterozygous variants: a pathogenic variant c.1204C>T (p.Arg402Trp) and a probable pathogenic variant c.853-26_854del (p.?). The c.1204C>T variant is one of the most frequent changes identified in patients of European origin with GA-1. The variant affects a conserved amino acid arginine and it is well-known that its replacement with tryptophan has deleterious consequences for structure and protein function. The c.853-26_854del mutation involves the deletion of 28 nucleotides from intron 8 and affects the canonical splice site -2 of exon 9 of the *GCDH* transcripts described in the *RefSeq* database. This variant has not been previously described in the healthy control population or in the literature associated with GA-1. 

A brain magnetic resonance imaging (MRI) scan was recommended (which was not performed because she became pregnant again) as well as a dietary record to assess protein intake and oral carnitine supplementation (50 mg/kg/day). She is currently nearing the end of her pregnancy and is clinically well.

### 3.3. Case 3

Female newborn from a controlled pregnancy with gestational diabetes. Eutocic vaginal delivery. Expulsion of meconium before 24 hours of life. Gestational age: 40 weeks. Birth weight: 3.190 g. Birth length: 51 cm. Cranial perimeter: 34 cm. Breastfeeding. Neonatal period without incidents. Family history: young parents of Moroccan origin. The mother was healthy, the father had type 2 diabetes mellitus. The parents had third-degree consanguinity. No previous miscarriages. The newborn had three healthy siblings who were 12, 9, and 6 years old. 

On the NBS, she presented with slightly increased levels of citrulline (61.8 µmol/; NV: 5.1–41.6) and high Cit/Phe (1.06; NV: 0.10–0.77). On a second DBS that was collected at 2 months of age, citrulline levels continued to be elevated (88.6 µmol/L; NV: 7.2–55.7) and high alanine levels were also observed for the first time (1138 µmol/L; NV: 191–940). At that time, the infant was asymptomatic with good weight development and the main suspicion was a transient elevation of citrulline or a mild form of citrullinemia type I. Therefore, it was decided that only biochemical monitoring would be performed. 

At 3 months of age, a plasma amino acid determination was performed, which revealed a moderately increased level of citrulline (127 µmol/L; NV: 2.8–34.2), normal levels of lysine (86 µmol/L; NV: 38–103), and a slightly elevated level of ammonia (92 µmol/L; NV: <60). At 7 months of age, ammonia levels had increased to 162 and AST was high (75 U/L; NV: 13–40). For this reason, it was decided to give the patient an appointment at our center for evaluation.

The patient came to our center for evaluation when she was 9 months old. At that time, the patient had had normal neurological development, meeting milestones appropriately. Growth had been normal and she ate a varied diet without vomiting or protein rejection. She weighed 9.5 kg and had not had any previous infections or notable symptoms. The physical examination was completely normal.

On the biochemical study carried out, compensated metabolic acidosis (pH 7.39, pCO_2_ 25 mmHg, and HCO_3_ 15 mmol/L) with normal glycemia (63 mg/dL) and high ammonia (179 µmol/L) was observed. She also had high levels of blood lactic acid (4.6 mmol/L; NV: <2.0), pyruvic acid (0.96 mg/dL; NV: 0.01–0.59), and serum CK (631 U/L; NV: 150–499). At that time, it was suspected that the origin of these abnormalities could be mitochondrial metabolopathy, especially the finding of pyruvate carboxylase deficiency, which is related to increased levels of citrulline on NBS.

The analysis of urinary organic acids revealed increased levels of 2-oxoglutaric acid (5112 mmol/mol crea; NV: 11–492), glutaric acid (31 mmol/mol crea; NV: 2–10), and 2-OH-glutaric acid (59 mmol/mol crea; NV: 5–48). Pyruvate carboxylase activity in cultured skin fibroblasts was normal (2319 pmol/min/mg protein; healthy control: 1933). 

During admission, a protein overload was performed in a single dose (2 g/kg), after which the patient presented with symptoms of clouding of consciousness, trembling, and vomiting. Plasma ammonium increased to >700. The hyperammonemia and neurological symptoms quickly yielded upon administration of carglumic acid.

A genetic panel of hereditary metabolic diseases revealed the homozygous pathogenic variant c.726G>A (p.Trp242*) in the *SLC7A7* gene, confirming that the patient’s disease was lysinuric protein intolerance (LPI). A subsequent analysis of urinary amino acids confirmed the expected high levels of lysine (813 µmol/L; NV: 15–95), which is the biochemical hallmark of this disease. At that time, when the patient was almost 1 year old, plasma citrulline (85 µmol/L; NV: 5–50) and alanine (1055 µmol/L; NV: 150–570) levels remained high.

Currently, the patient is progressing well and receives a protein-restricted diet of 1 g/kg/day with protein- and amino acid-free infant formula, L-citrulline 1g/day, and carnitine 30 mg/kg/day. Introducing phenylbutyrate will be considered if necessary. Over long-term follow-up, the onset of autoimmune symptoms, dyslipidemia, and impaired cognitive development will be closely monitored.

### 3.4. Case 4

Male newborn from a controlled pregnancy without incidents. Eutocic vaginal delivery. Expulsion of meconium before 24 hours of life. Gestational age: 37 weeks. Birth weight: 3.050 g. Birth length: 52 cm. Breastfeeding. Family history: healthy 33-year-old mother and 35-year-old father. The mother had seven pregnancies with five children who are 17, 13, 6, 3, and 1 years old and one voluntary abortion. There was no consanguineous relationship between the parents or family history of interest. Expanded NBS was available for the three youngest siblings—their NBS profiles of amino acid and acylcarnitines were normal.

On the NBS, he presented with a slight elevation of C3/C2 (0.18; NV: <0.17) with normal levels of C3 (3.40 µmol/L; NV: 0.69–3.87). On a second DBS that was collected at 15 days of age, C3/C2 level remained slightly elevated (0.17), suggesting a possible maternal vitamin B_12_ deficiency or, less probably, methylmalonic acidemia.

On the biochemical study carried out on the newborn and his mother, the suspected maternal vitamin B_12_ deficiency was confirmed. In the newborn, plasma C3 levels were high (0.95 µmol/L; NV: 0.15–0.89), serum vitamin B_12_ was normal (616 pg/mL; NV: 211–911), plasma total homocysteine was high (12.4 µmol/L; NV 0–11), and urinary methylmalonic acid was high (14 mmol/mol crea; NV: 0–11). In the mother, serum vitamin B_12_ was low (198 pg/mL) and plasma total homocysteine was very high (49.9 µmol/L). The complete blood count of both mother and child revealed no significant findings. An additional autoantibody study was performed on the mother to rule out pernicious anemia as the cause of the deficiency; it was negative. As both were asymptomatic, they were prescribed oral vitamin B_12_ supplementation and were discharged.

Two years later, the mother was asked to return to the newborn screening laboratory for a reevaluation because the origin of the vitamin B_12_ deficiency had not been clarified and she had strikingly high levels of homocysteine at the time of diagnosis. The new biochemical study carried out found very high levels of homocysteine (50 µmol/L), with normal serum vitamin B_12_ levels (325 pg/mL) and urinary methylmalonic acid levels (1 mmol/mol crea). The serum folic acid level was slightly low (2.4 ng/mL; NV: 3.1–17.5) and the C-reactive protein level was very high (30.8 mg/L; NV: <5.0). Having ruled out vitamin B_12_ deficiency as the cause of the hyperhomocysteinemia, we considered carrying out a genetic panel of homocystinurias. Upon re-interviewing the patient, it was discovered that her maternal grandmother had had an episode of thrombosis and her maternal aunt had had a stroke at 60 years of age.

The genetic study identified a heterozygous pathogenic variant in the *MMACHC* gene c.271dupA (p.Arg91Lysfs*14), which is associated with cobalamin C deficiency (clb C, methylmalonic aciduria, and hyperhomocysteinemia) and a homozygous functional polymorphism c.665C>T (p.Ala222Val) in the *MTHFR* gene; this is associated with mild-to-moderate hyperhomocysteinemia. No other pathogenic variants were identified in the exonic sequence of *MMACHC*. To rule out the presence of other variants affecting the expression of *MMACHC* (e.g., epimutation, variants in the untranslated region (UTR) sequences, etc.), we performed RNA analysis. The results showed the presence of the pathogenic variant c.271dupA in heterozygosity, suggesting that the patient was in fact a cblC carrier.

Currently, the patient is well and is being treated with oral vitamin B_12_ and folic acid supplements. Other investigational genetic studies are being carried out to determine if there is another alteration that may better explain the levels of homocysteine that she presented with.

## 4. Discussion

### 4.1. Case 1

Expanded NBS for amino acid disorders using tandem mass spectrometry includes the possibility of determining arginine levels, thus allowing for the detection of increased risk for arginase-1 deficiency [13]. Our patient presented with slightly elevated levels of arginine on the NBS results. However, the different diagnostic tests performed on the patient ruled out arginase-1 deficiency. 

This case illustrates the importance of continuing to investigate an unexplained finding in order to contribute to advancing the knowledge of hereditary metabolic diseases. It also highlights the role of next generation sequencing (NGS) techniques that allow for the identification of the molecular defect and the possibility, as in this case, of discovering a new disorder through an incidental finding on an expanded NBS.

To date, this is the only case report of a defect in human CAT-2. The characteristic biochemical profile of human CAT-2 deficiency in this patient consisted of an elevation in arginine, ornithine, and lysine levels in plasma and urine. The natural history of the disorder over long-term follow-up is uncertain, though a low-protein diet is necessary to control amino acid levels. This defect can potentially be detected through expanded NBS programs. NBS centers should be aware of this disorder since it can be incidentally detected while screening for arginase-1 deficiency [12].

### 4.2. Case 2

This case reflects the role of NBS in the potential detection of undiagnosed maternal metabolic diseases. One of the most representative examples is maternal GA-1, which mimics a carnitine transporter defect in the newborn. This special finding is well known and there are a few cases reported in the literature. Some mothers were symptomatic and others asymptomatic at the time of diagnosis [14,15,16,17]. Curiously, levels of C5DC in the newborn are normal and what is usually observed is a secondary depletion of free carnitine levels [17]. Low levels of free carnitine on NBS have also been described in maternal carnitine transporter deficiency and maternal MCAD deficiency [18], which thus entails making a differential diagnosis among these disorders. In our experience, approximately half of the suspected cases of carnitine transporter defect in the newborn are of metabolic maternal origin. Interestingly, plasma total carnitine was significantly lower in other maternal conditions than in maternal carnitine transporter deficiency [16]. 

Maternal GA-1, in contrast to other metabolic disorders, does not seem to affect the health of the fetus, though it is not known if fetal maturation could be influenced by low levels of maternal carnitine [15,19]. The phenotypic spectrum of GA-1 is broad, ranging from severely affected individuals to asymptomatic adults [20,21]. Regarding the new variant identified in our patient, the correlating clinical phenotype that it can present as is unknown, although other similar splicing variants in this gene have previously been described as pathogenic.

In a Portuguese series of GA-1 mothers detected through NBS, the presence of macrocephaly was a very common finding, despite a lack of neurological symptoms (unpublished data). At diagnosis, some of these mothers complained of intermittent fatigue [17], although this is a very unspecific symptom that may be not related to GA-1 or secondary carnitine deficiency. 

Our newborn’s DBS profile completely mimicked a carnitine transporter deficiency. However, two slightly elevated ratios (C5DC/C8 and C5DC/C16) were detected, which could lead to suspecting the presence of maternal GA-1, since this finding is not found in neonatal or maternal carnitine deficiencies. It is necessary to review more NBS profiles of newborns from GA-1 mothers to determine if this is a common finding or not.

### 4.3. Case 3

This is an unusual case of LPI detected on NBS through slightly elevated levels of citrulline that initially led to suspicion of mild citrullinemia type I or pyruvate carboxylase deficiency. This case shows us how difficult it can be to reach a diagnosis when the biochemical profile observed on NBS has not previously been associated with a metabolic disease but rather guides us towards other diseases. With a simple laboratory test (urinary amino acid determination), the diagnosis could have been made. Nevertheless, an NGS was required in order to identify the specific disease. It should be noted that during the diagnostic evaluation, the patient presented with persistent mild–moderate hyperammonemia and developed an episode of severe hyperammonemia in response to protein overload, which is characteristic of LPI. 

High levels of citrulline on NBS have not been previously associated with LPI. However, some patients of Malaysian origin have been reported to have slightly elevated plasma citrulline levels on biochemical studies [22,23]. The persistence of citrulline elevation in our patient, together with these previously reported cases, lead us to think that increased citrulline values could be associated with LPI, rather than being a coincidental finding. This may also be true in the case of high alanine levels, which could be related to lactic acidosis. Our hypothesis as to why no other cases of LPI detected through NBS have been reported is that citrulline levels may be slightly elevated, and these results are usually considered transitory conditions or NBS false positives. It would be very illuminating to retrospectively review the NBS citrulline levels of patients clinically diagnosed with LPI. Until this hypothesis can be ruled out, we think it is preferable to include this disease in the differential diagnosis of newborns with high citrulline levels on NBS and to measure urinary amino acids as part of the diagnostic approach.

### 4.4. Case 4

Maternal vitamin B_12_ deficiency is a relatively common finding on expanded NBS, especially in exclusively breastfed infants. If this condition is suspected, it is advisable to carry out a biochemical study of the newborn and the mother to identify the cause of the deficiency, which is generally of maternal origin (vegetarianism, unknown pernicious anemia, and nutritional or digestive problems are the most frequent reasons).

The case presented is an example of a mild vitamin B_12_ deficiency detected on NBS. However, in addition to this condition, the mother presented with plasma total homocysteine levels that were not consistent with the degree of deficiency. For this reason, she was invited to return 2 years later. At that point, she no longer had a vitamin B_12_ deficiency, which in her case could probably have originated or been aggravated by multiparity, but her homocysteine levels were strikingly high. 

The presence of the homozygous polymorphism c.665C>T on the *MTHFR* gene has been associated with hyperhomocysteinemia and a risk of thrombosis, depression, and carotid atherosclerosis [24,25,26]. It is possible that elevated levels of C-reactive protein reflect a proinflammatory status. However, we do not know if this polymorphism alone can explain such high homocysteine levels. Furthermore, it is unknown if the pathogenic variant c.271dupA identified on the *MMACHC* gene may contribute to some extent as well. Unfortunately, it is quite common for many positive NBS cases not to be completely closed. Continuing patient follow-up is necessary in order to assess her response to folic acid and vitamin B_12_ oral supplementation, to monitor her C-reactive protein levels, and to complete ongoing research studies.

## 5. Conclusions

Each defect detected through expanded NBS may be an unusual case. A careful study of incidental findings is an opportunity to discover conditions whose treatment may have a positive impact on the health of newborns and their families. Adequate early detection of these disorders constitutes a great challenge for laboratory professionals whereas optimal clinical follow-up and therapeutic management represent a challenge for pediatric specialists in hereditary metabolic disorders. Expanded NBS for inborn errors of metabolism is a promising field of targeted metabolomics, and NGS technology has gradually become of great importance in the field of genetic diagnosis. The possibility of performing an increasingly precise NBS is already a reality that is allowing us to expand our knowledge of these diseases. 

## Figures and Tables

**Table 1:** Dried blood spot (DBS) acylcarnitine profile in the newborn and his mother.

Marker	Newborn DBS Results	Maternal DBS Results	Reference Values for Newborns < 7 days (µmol/L)
Free carnitine (C0)	2.50	1.88	11.0–50.0
Acetylcarnitine (C2)	5.11	2.04	8.6–52.0
Propionylcarnitine (C3)	0.33	0.17	0.69–3.87
Butyril/isobutyrilcarnitine (C4)	0.03	0.02	<0.64
Isovalerylcarnitine (C5)	0.06	0.05	<0.50
Glutarylcarnitine (C5DC)	0.08	0.80	<0.19
Hexanoylcarnitine (C6)	0.04	0.03	<0.15
Octanoylcarnitine (C8)	0.01	0.01	<0.17
Decanoylcarnitine (C10)	0.04	0.01	<0.20
Miristoylcarnitine (C14)	0.04	0.02	<0.58
Palmitoylcarnitine (C16)	0.60	0.13	0.86–5.85
Stearoylcarnitine (C18)	0.20	0.10	0.34–1.83
